# *Shewanella haliotis* Associated with Severe Soft Tissue Infection, Thailand, 2012

**DOI:** 10.3201/eid1906.121607

**Published:** 2013-06

**Authors:** Kittiyod Poovorawan, Tanittha Chatsuwan, Narisorn Lakananurak, Jira Chansaenroj, Piyawat Komolmit, Yong Poovorawan

**Affiliations:** Chulalongkorn University, Bangkok, Thailand

**Keywords:** Shewanella haliotis, liver transplant, soft tissue infection, septic shock, bacilli, bacteria, antibiotic, antibacterial, antimicrobial, immunocompromised, immune compromised, tropical

**To the Editor:** Marine bacteria of the family *Shewanellaceae*, genus *Shewanella*, are gram-negative, motile bacilli that grow aerobically or anaerobically and produce hydrogen sulfide (*1*). Organisms belonging to a *Shewanella* species were first isolated in 1931 by Derby and Hammer from dairy products and classified as *Achromobacter putrefaciens* (*2*). Members of *Shewanella* species usually are found in marine environments in warm climates or during summer in temperate climates (*3*). In humans, most *Shewanella* species infections occur in skin and soft tissues (*4*). One species (*S. algae*) and possibly a second (*S. putrefaciens*) have been isolated from human samples on multiple occasions (*5*). A third species, *S. haliotis*, was implicated in human infections during 2010 (*6*) and *S. xiamenensis* was reported as the fourth infectious species among humans during 2011 (*7*). *S. haliotis* is a novel bacterial species that was isolated from the gut microflora of abalones (*Haliotis discus hannai*) in 2007 (*8*). We report the second description, to our knowledge, of *S. haliotis* involved in human disease.

In September 2012, a 52-year-old woman, living in Bangkok, Thailand, was hospitalized after experiencing drowsiness for 2 hours. She had a low-grade fever, chills, and swelling, erythema, and tenderness in her left leg. During the previous week, she had handled fresh seafood in a market and had eaten cooked mackerel. She denied having eaten uncooked food or wading into flooded areas or the sea. She had undergone orthotopic liver transplantation 6 months previously to excise hepatocellular carcinoma related to Child-Pugh class C hepatitis C cirrhosis; since that procedure, she had been under treatment with immunosupressive drugs. She also had diabetes, hypertension, and nephrotic syndrome. Physical examination revealed that in addition to above-named symptoms, multiple blisters were noted (Figure, panel A). Her oral temperature was 37.8°C, blood pressure 80/40 mm Hg, pulse was 110 bpm, and respiratory rate was 24 breaths/minute. A complete blood count showed a leukocyte count of 2,250 cells/μL (91.2% neutrophils). Despite adequate rehydration, monitored by central venous pressure, the patient required norepinephrine to stabilize her vital signs. The clinical diagnosis of her condition was septic shock with suspected necrotizing fasciitis. 

**Figure:**
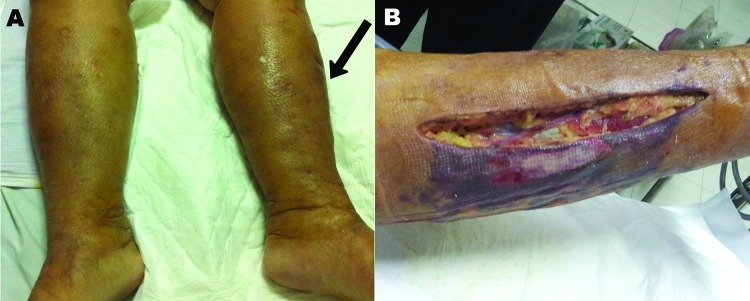
*Shewanella haliotis* severe soft tissue infection of woman in Thailand, 2012. The patient sought treatment for painful erythematous swelling of the left leg. A) Arrow indicates affected area. B) Postsurgical fasciotomy wound with necrotic tissue.

After tissue and blood samples were collected and submitted for microbiological analysis, shock resuscitation and an emergency fasciotomy (Figure, panel B) were performed, and antimicrobial drug treatment with meropenem and vancomycin was started. Surgeons did not confirm the suspected necrotizing fasciitis. Two sets of blood cultures and fluid culture sampled from the left leg identified *S. algae* by conventional biochemical methods. The MICs of antimicrobial drugs were determined by Etest (bioMérieux, Solna, Sweden). This strain was susceptible to ciprofloxacin (0.25 mg/L), piperacillin-tazobactam (1.0 mg/L), ceftriaxone (1.0 mg/L), and meropenem (0.38 mg/L). The patient had fever for the first 2 days of hospitalization. After 2 weeks of treatment, the antimicrobial drug was switched to oral ciprofloxacin; treatment was continued after dressing and debridement of the fasciotomy wound.

The organism produced yellowish-brown mucoid colonies on sheep blood agar and chocolate agar after 18 hours of incubation at 35^°^C under CO_2_ atmosphere. MacConkey agar showed non–lactose-fermenting colonies that were oxidase-positive, motile, and produced hydrogen sulfide on triple sugar iron agar. Growth at 42^o^C with 6.5% NaCl suggested that this organism was *S. algae*. Because phylogenetically related *Shewanella* species may be misidentified by routine biochemical tests, the strain was confirmed by using 16S rRNA gene sequencing.

Molecular characterization of 16S rRNA gene sequencing was performed by using PCR with *Shewanella* species consensus primers (Technical Appendix Table) and direct sequencing from PCR product (JX968803). Phylogenetic analysis of the 16S rRNA gene sequence showed clustering with *S. haliotis* (NR_044134^T^) and 99.9% similarity and 1 base difference (online Technical Appendix Figure). By using BLAST (http://blast.ncbi.nlm.nih.gov/Blast.cgi) analysis, JX968803 showed the closest match (99.9%; 1 base difference) with *Alteromonadaceae bacterium* PH39 (AF513471).

The strain was confirmed as *S. haliotis* by using additional biochemical tests and API 20 NE System (bioMérieux, Durham, NC, USA). It was positive for ornithine decarboxylase, gelatinase, reduction of nitrates to nitrites, tolerance to 6% NaCl, and assimilation of caprate and malate, but negative for citrate utilization, arginine dihydrolase, lysine decarboxylase, urease, indole production, assimilation of mannose, glucose, arabinose, mannitol, maltose, adipate, and acidification of glucose. This strain was resistant to polymyxin B (300 µg/disc). 

More than 50 species of *Shewanella* have been reported. The route of *Shewanella* infection is associated with direct contact with the organism through seawater or ingestion of raw seafood (*9*). Japan reported 1 case of *S. haliotis* infection in an elderly patient in whom *Vibrio vulnificus* infection was initially suspected (*6*), and various clinical manifestations of *S. algae* infection have been reported (*5*). Community- and hospital-acquired infection with *Shewanella* species from contaminated medical devices have also been reported (*10*). *S. haliotis* and *S. algae* are closely related organisms; discriminating between them on the basis of biochemical tests is difficult. Molecular characterization of 16S rRNA gene sequencing can be used to differentiate the 2 species. In summary, this case suggests that immune-compromised persons in tropical climates could be susceptible to *S. haliotis* soft tissue infection in the absence of typical exposures.

Technical Appendix Specific *Shewanella* consensus primer sets for 16S rRNA gene sequencing and phylogenetic analysis.
