# Supplementary material for: Shewanella haliotis Associated with Severe Soft Tissue Infection, Thailand, 2012
Source: Emerg Infect Dis. 2013 Jun;19(6):1019–21. doi: 10.3201/eid1906.121607 (PMC3713828; doi:10.3201/eid1906.121607)
Supplement: Technical Appendix — Specific Shewanella consensus primer sets for 16S rRNA gene sequencing and phylogenetic analysis. [file 12-1607-Techapp-s1.pdf]

Article DOI: http://dx.doi.org//10.3201/eid1906.121607

Shewanella haliotis Associated with Severe Soft Tissue Infection, Thailand, 2012

Technical Appendix

Specific *Shewanella* Consensus Primer Sets for 16S rRNA Gene Sequencing and Phylogenetic Analysis

Technical Appendix Table. Specific *Shewanella* consensus primer sets for 16S rRNA gene sequencing performed during investigation of soft tissue infection, Thailand, YEAR**.**

Round	Primer name	Sequence (5'→3')	Product (bp)
1	shew16s_99F	CGA GCG GCG GAC GGG TGA G	1,327
	shew16s_1426R	CCA CTC CCA TGG TGT GAC G	
2	shew16s_349F	GGA GGC AGC AGT GGG GAA	1,077
	shew16s_1426R	CCA CTC CCA TGG TGT GAC G	

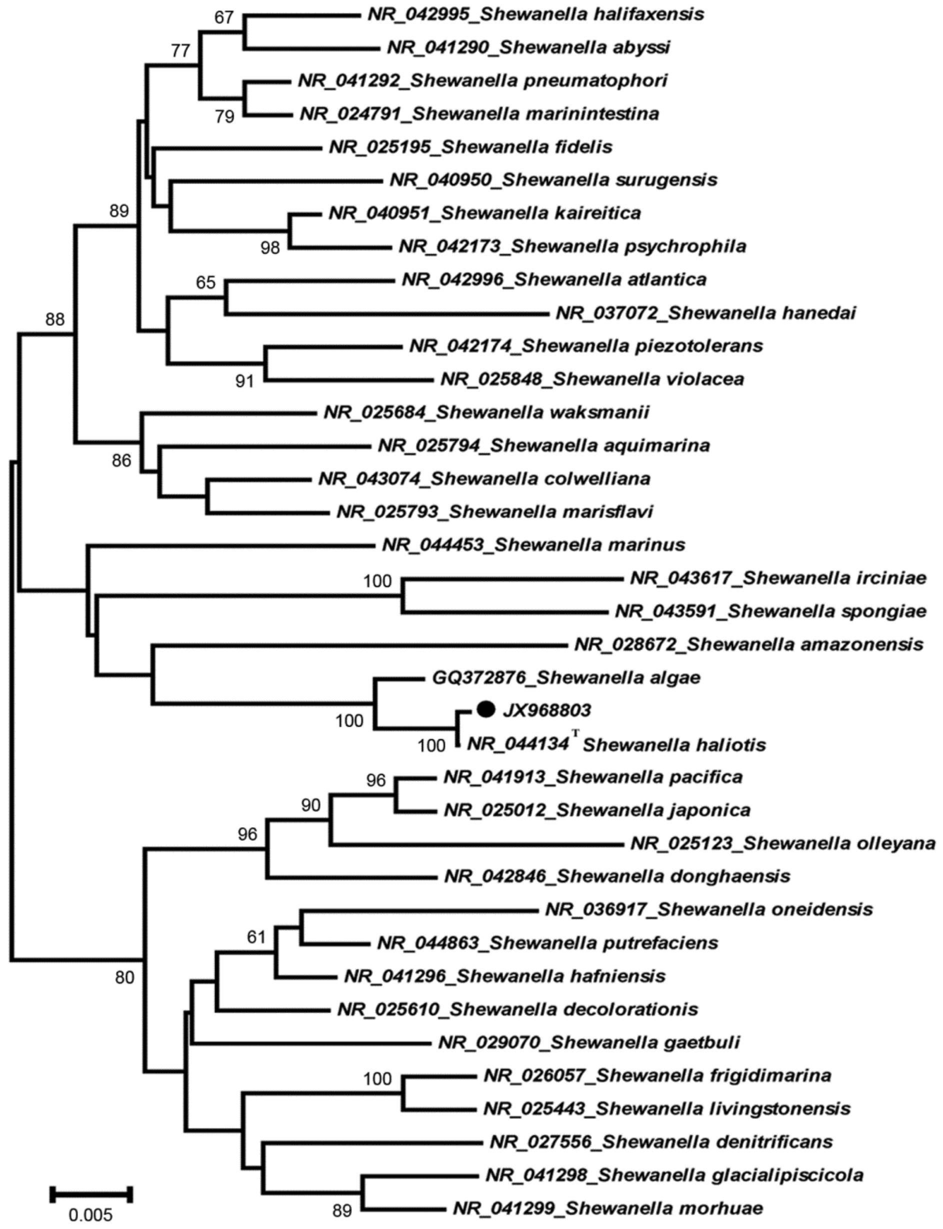

Technical Appendix Figure. Phylogenetic analysis of *Shewanella* spp. 16S rRNA gene sequencing (1,077 base pairs). Closed circle indicates the strain in this study compared with the *S. haliotis* prototype species (NR_044134^{T}) and other representative species. Numbers at branch nodes are bootstrap values. Scale bar represents number of nucleotide substitutions per site.
